# Progress in Nano-Biosensors for Non-Invasive Monitoring of Stem Cell Differentiation

**DOI:** 10.3390/bios13050501

**Published:** 2023-04-26

**Authors:** Min-Ji Kang, Yeon-Woo Cho, Tae-Hyung Kim

**Affiliations:** School of Integrative Engineering, Chung-Ang University, 84 Heukseuk-ro, Dongjak-gu, Seoul 06974, Republic of Korea

**Keywords:** stem cell differentiation, biosensing, nano- and micromaterials

## Abstract

Non-invasive, non-destructive, and label-free sensing techniques are required to monitor real-time stem cell differentiation. However, conventional analysis methods, such as immunocytochemistry, polymerase chain reaction, and Western blot, involve invasive processes and are complicated and time-consuming. Unlike traditional cellular sensing methods, electrochemical and optical sensing techniques allow non-invasive qualitative identification of cellular phenotypes and quantitative analysis of stem cell differentiation. In addition, various nano- and micromaterials with cell-friendly properties can greatly improve the performance of existing sensors. This review focuses on nano- and micromaterials that have been reported to improve sensing capabilities, including sensitivity and selectivity, of biosensors towards target analytes associated with specific stem cell differentiation. The information presented aims to motivate further research into nano-and micromaterials with advantageous properties for developing or improving existing nano-biosensors to achieve the practical evaluation of stem cell differentiation and efficient stem cell-based therapies.

## 1. Introduction

Stem cells can differentiate into specific cell subtypes, which has resulted in the development of tissue engineering and regenerative medicine [1,2]. Due to stem cells’ ability to produce cells in vitro that are associated with the physiological functions of specific tissues, stem cell therapy has emerged as a potential solution for many diseases that are difficult to treat with conventional chemotherapy over the past few decades [3,4,5,6]. There have been 40,183 research papers about stem cell therapy published between 1971 and 2021; many of these studies demonstrated its clinical potential. However, the only stem cell therapy approved by the United States Food and Drug Administration to date is haematopoietic (or blood) stem cell transplantation [7,8,9,10].

There are many challenges in the development of stem cell therapy, including low differentiation efficiency, differentiation into undesired cell subtypes, carcinogenesis, and post-transplant inflammatory response [11,12]. Therefore, many stem cell differentiation studies have been conducted to (i) understand the developmental stages of stem cell differentiation, (ii) control stem cell behaviour in vitro, and (iii) enhance stem cell differentiation efficiency [13,14,15,16]. Consequently, a need for measuring stem cell differentiation using a variety of analytical methods has arisen. These techniques include polymerase chain reaction (PCR), immunocytochemistry, flow cytometry and Western blot (WB), which have been widely used with biomarkers, such as proteins, ribonucleic acid (RNA) and deoxyribonucleic acid (DNA) [17,18,19,20]. However, these techniques are destructive, laborious, and costly; therefore, they are inappropriate for the quantitative and qualitative analysis of differentiated cells prearranged in therapeutic transplantation [21]. Hence, non-destructive and real-time monitoring of cell differentiation is necessary for efficient stem cell therapy. Many biosensing and nanotechnology methods have been proposed for the non-invasive monitoring of stem cell differentiation, such as impedance and Raman spectroscopy, deep learning-based approaches, electrochemical immunoassay biosensors, and electroluminescence [22,23,24,25,26,27,28,29,30,31,32,33]. In particular, electrochemistry-based sensing methods, such as impedance spectroscopy and electroluminescence, have been demonstrated as analytical techniques that selectively detect target materials through electrical signals generated from the redox reaction of analytes. These techniques have the following advantages: (i) facile, (ii) inexpensive, (iii) simple, portable analytical devices, and (iv) non-invasive [34,35,36,37,38]. Similarly, optical sensing methods, such as fluorescence, near-infrared (NIR), and Raman spectroscopy, can selectively detect the optical properties or signals of target materials. These methods have the following advantageous features: (i) high selectivity, (ii) flexibility, and (iii) non-invasive [39,40,41,42,43,44].

The medium for in vitro stem cell cultivation contains cells with many organelles, but also several types of proteins, small molecules, and other chemicals; this means that the analytical conditions for cell-based sensing are highly complex [41,45]. Therefore, improving sensing performance, including sensitivity and selectivity towards target analytes, is essential for the accurate and sensitive label-free monitoring of stem cell differentiation with electrochemical or optical-based sensors. More specifically, a highly sensitive sensing capability for differentiation-associated targets is required to quantitatively analyse how much differentiation was induced from the stem cells in real-time. In addition, to qualitatively analyse whether specific differentiation into desired cell subtypes has been induced during stem cell differentiation, selectivity for the analytes is a key indicator.

Various nano- and micromaterials have been used to modify sensor surfaces to improve performance, including sensitivity, selectivity, and reliability [44,46]. For instance, highly conductive metal nanomaterials, such as gold nanoparticles (AuNPs) and silver nanoparticles (AgNPs), and carbon-based conductive materials, such as graphene oxide (GO), have excellent electrical or electrochemical properties and are widely used in electrochemical sensors [47,48]. In addition, three-dimensional (3D) micromaterials, such as microelectrode assays and microfluidics, have been used to improve electrochemical sensors’ performance by increasing the active surface area [49,50,51]. In the case of Raman spectroscopy-based sensors, two-dimensional (2D) or 3D combinations of metal nanoparticles with good optical properties and carbon-based conductive materials have been used to improve the sensitivity [52,53]. However, each electrochemical and optical-based sensor’s sensing mechanism is different; therefore, the strategies for improving the sensing performance and capabilities and the techniques for sensor surface modification are different.

**Figure 1:**
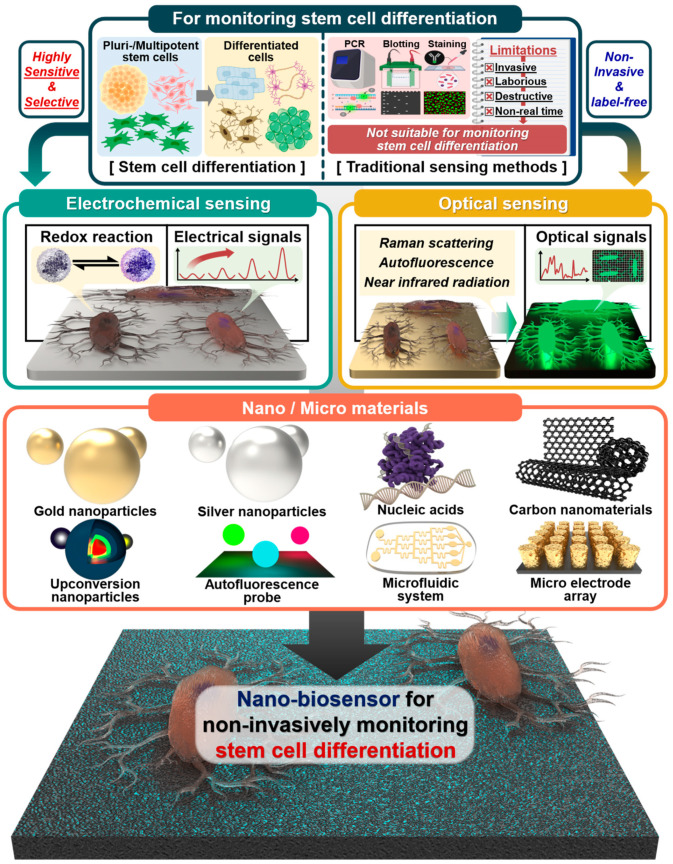
Schematic illustration of traditional sensing and nano- and micromaterial-based methods for monitoring stem cell differentiation. Created with BioRender.com.

This review highlights and compares recent studies on non-invasive and real-time monitoring of stem cell differentiation, including neurogenesis, cardiomyogenesis, osteogenesis, and adipogenesis (Figure 1). In addition, various biosensors fused with specific analysis technology, such as electrochemistry and optical sensing, and various nano- and micro materials, such as AuNPs, AgNPs, upconversion nanoparticles (UCNPs), autofluorescence probes, nucleic acids, microfluidic systems, and microelectrode arrays, are reviewed and compared (Table 1).

## 2. Electrochemical Sensors

### 2.1. Gold Nanoparticle-Based Electrochemical Sensors

Gold’s conductivity is 4.11 × 10^7^ S/m, which is highly favourable for electrochemical sensors [54]. Moreover, gold is colloidal in AuNPs, which has various advantageous features for electrochemical sensors [55,56,57,58,59,60,61,62,63]. For example, AuNPs are easy to synthesise and can be conjugated with multiple biomolecules, such as protein ligands, nucleic acids, and antibodies, which can enforce the intrinsic properties of the AuNPs [64,65,66]. Moreover, it has been reported that AuNPs are non-cytotoxic, cell-friendly materials with great potential for sensing biomolecules in a cell-based environment and for cell cultivation platforms [67,68,69,70,71,72]. Therefore, AuNPs have been widely used to develop electrochemical sensors with new nanostructures or modify existing electrochemical electrode surfaces at the nanoscale.

Many studies have described AuNP-based electrochemical sensors capable of sensing stem cell differentiation. For example, Suhito et al. developed an electrochemical AuNP-based electrochemical nano-biosensor to identify the differentiation of embryonic stem cells (ESCs) [59]. To fabricate this sensor, AuNPs were densely deposited on a transparent indium–tin oxide-coated glass electrode through electrochemical deposition. The study’s cell detection results using differential pulse voltammetry (DPV) showed that the undifferentiated ESCs generated relatively strong electrochemical signals compared with differentiated ESCs-derived endothelial cells. Interestingly, this sensor could sensitively measure ESCs based on the high electrical conductivity of gold, detecting at least 12,500 cells on one platform. Moreover, it was shown that this sensor could ensure ESCs’ adhesion and long-term cell growth, suggesting its application as an ESCs’ cultivation platform and a platform for electrochemically monitoring various stem cell differentiation.

In another study, Lee et al. described a AuNP-based nano-biosensor for non-invasive, real-time monitoring of the osteogenesis of mesenchymal stem cells (MSCs) [73]. Specifically, this sensor comprised a 3D AuNP-based nanoarray; the surface of the gold nanoarray was modified with GO. This nanostructure efficiently increased gold’s electrical conductivity and electron transfer rate through GO modification, allowing the detection of p-aminophenol (PAP) produced by the enzymatic reaction occurring in MSCs’ osteogenesis. Furthermore, this study observed that this nanoarray provided physicochemical cues beneficial to cellular adhesion and osteogenic differentiation. As a result, this gold-based electrochemical sensing platform detected the anodic signals of PAP using cyclic voltammetry (CV), quantitatively monitoring differentiation during 3 weeks of osteogenesis.

**Figure 2:**
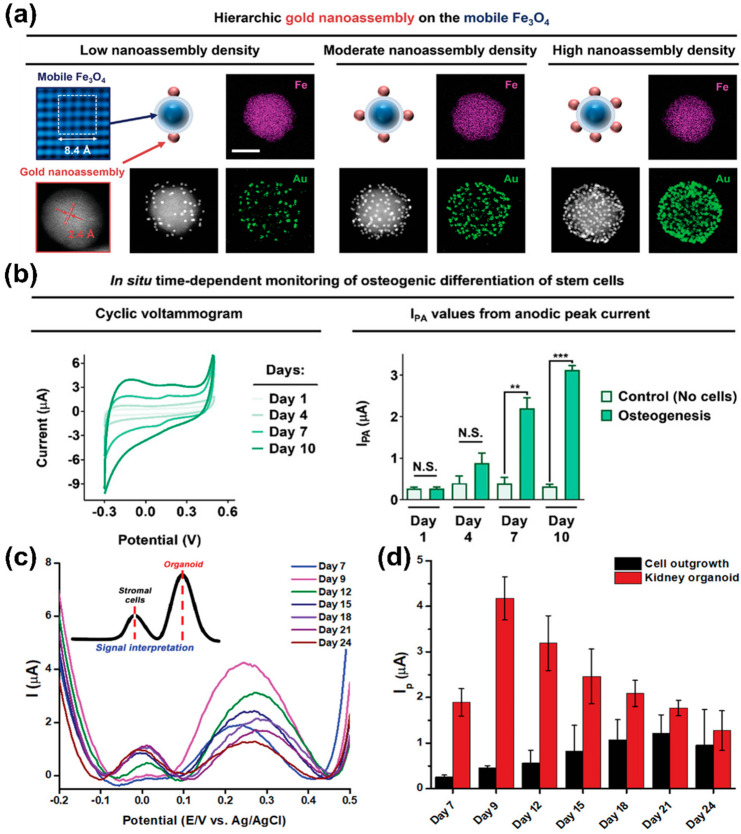
Gold nanoparticle-based electrochemical sensors. (**a**) Characterisation of gold nanoassembly and magnetic nanoparticles. (**b**) Time-dependent monitoring of osteogenesis using the gold nanoassembly-based electrochemical sensors. (**c**,**d**) DPV results of iPSCs differentiation and organoid generation on gold-based electrochemical electrode. Reprinted with permission from [74]. Copyright 2021, Wiley Online Library; Reprinted with permission from [75]. Copyright 2022, Wiley Online Library. AuNPs, gold nanoparticles; DPV, differential pulse voltammetry; Fe_3_O_4_, iron (II, III) oxide; iPSCs, induced pluripotent stem cells. N.S. indicates “not significant.” ** *p* < 0.01, and *** *p* < 0.001.

In 2021, a AuNP-based sensing platform was developed that monitored the differentiation of stem cells and controlled their cell differentiation by regulating cellular adhesion (Figure 2a,b) [74]. This sensor was based on a nanoassembly in which AuNPs and Arg-Gly-Asp peptide (RGD) ligands were conjugated on the surface of magnetic iron (II, III) oxide (Fe_3_O_4_) nanoparticles. Specifically, the magnetite mediated the control of falling and rising ligand movements via linker compression and stretching, thereby regulating MSCs’ integrin expression pattern, cellular adhesion, and consequent osteogenic differentiation. Additionally, due to the high electrical conductivity of the AuNP nanoassembly-based sensor, osteogenic differentiation could be monitored by sensitively measuring PAP’s redox using CV.

In 2022, a AuNP-based electrochemical nano-biosensor capable of sensing the generation process and maturity of kidney organoids produced through the differentiation of induced pluripotent stem cells (iPSCs) was developed (Figure 2c,d) [75]. Moreover, the variations of organoids led to the need for a non-destructive evaluation of their maturity [76,77,78]. Therefore, this sensor was developed to assess the iPSCs differentiation into kidney organoid by sensitively detecting the electrochemical signals originating from the organoids through a gold film structure on which there was electrochemically deposited AuNPs. Interestingly, while monitoring the kidney organoid generation on this sensor, two peaks were detected in the DPV results. Specifically, it was confirmed that the first peak corresponded to cell outgrowth, while the second peak differed depending on the maturity of kidney organoids. A strong second peak was observed for organoids with distinct tubular structures. These results demonstrate that this electrochemical sensor could detect the successful production of kidney organoids in a label-free, non-destructive manner.

In general, studies have shown that AuNPs can be applied to develop electrochemical sensors that sensitively detect the target analytes involved in stem cell differentiation because of AuNPs’ excellent electrical conductivity, versatility, and ease of synthetic manipulation with various biomolecules and nanomaterials.

### 2.2. Nucleic Acid-Based Electrochemical Sensors

Nucleic acids, including DNA and RNA, are biopolymers composed of nucleotide units [79]. Nucleic acid molecules have specific nucleotide sequences, which comprise of the bases of adenine, thymine, cytosine, and guanine, that can bind strongly with complementary base-pair sequences. The intrinsic properties of nucleic acids have the potential as nanomaterials for biosensors [74,80,81,82,83], as they impart high selectivity in terms of the capability to detect target molecules selectively. In addition, nucleic acid-based aptamers can be developed as ligands for target materials through the systematic evolution of ligands by exponential enrichment technology [84,85]. These aptamers can be developed faster and cheaper than antibodies. Moreover, aptamers have unique 3D structures (e.g., loop, stem, quadruplex, bulge, hairpin, and pseudoknot) through the nucleotide sequence alignment of the nucleic acids; this allows the aptamers to bind more strongly and selectively to the target [86,87,88]. In addition, nucleic acid materials can be chemically conjugated to fluorescent or electrochemical probes, giving a stronger signal and higher affinity toward analytes.

Park et al. [89] described a microelectrode based on carboxylated polypyrrole nanotubes conjugated with aptamers capable of evaluating the neuronal maturation of neurons. These authors showed that the aptamer-based sensor could sensitively and selectively measure dopamine (DA) exocytosis as a neuronal function. To improve the sensor’s performance, the DA sensitivity according to the carboxylated polypyrrole nanotube diameter was first analysed. Then, the optimised aptamer-based sensor was evaluated for DA-sensing performance using amperometry, which showed an excellent limit of detection (LOD) of 100 pM. Furthermore, the sensor could electrochemically distinguish DA in the presence of other neurotransmitters, such as norepinephrine, serotonin, and phenethylamine. This study demonstrated that the developed sensor could electrochemically detect exocytotic DA released from neuronal cells due to DA’s high sensitivity and selectivity. The study’s results suggested the possibility of aptamer-based electrochemical sensors to monitor the neural differentiation process of stem cells.

A nucleic acid-based electrochemical sensor capable of monitoring cardiomyocyte differentiation was reported in 2021 [90]. This nucleic acid-based sensor contained hybrid materials, including short DNA domains and peptide motifs that bind complementarily to cardiomyocyte-specific regulatory proteins. Notably, this sensor showed a low level of LOD of 0.42 pg/mL. In addition, the sensor selectively detected electrochemical signals from cardiac troponin (cTnl) as a target molecule in the presence of other proteins, including human serum albumin and human brain natriuretic peptide. Due to the sensor’s high affinity and sensitivity to cTnl, it was possible to determine the cTnl expression level through electrochemical signals measured from cardiomyocytes differentiated from MSCs. In addition, the electrochemical signal for cTnl obtained while monitoring the cardiomyocyte differentiation process was consistent with the result of flow cytometry, validating the high reliability of this sensor.

**Figure 3:**
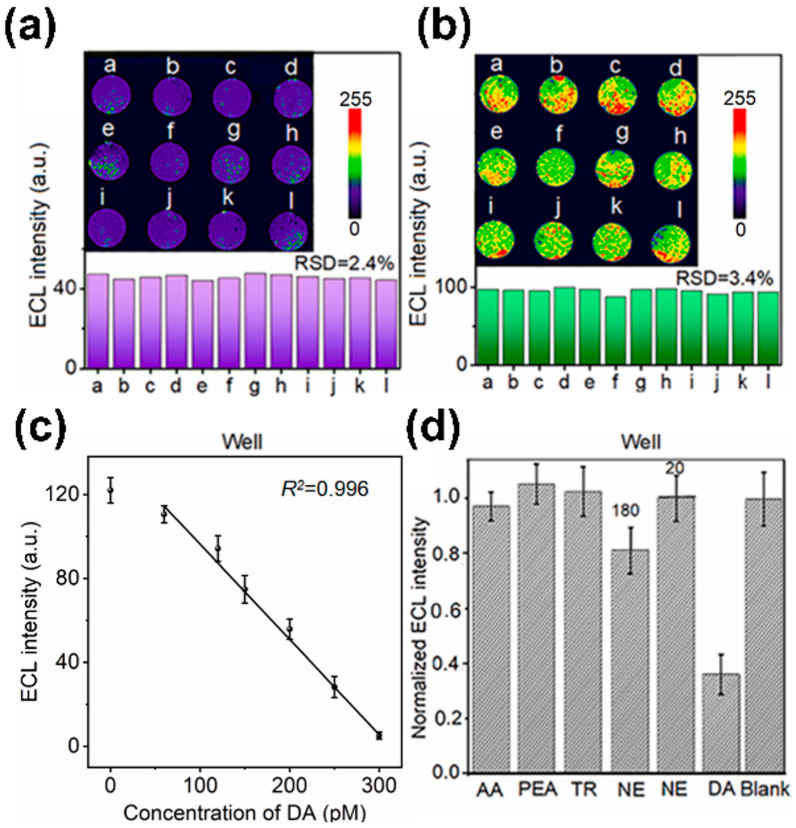
Aptamer-based electrochemical sensors. (**a**,**b**) ECL images of neurons injected in the micro-well and DA aptamer-based electrochemical sensor. (**c**,**d**) Analysis of DA sensing capability of micro-well and DA aptamer-based electrochemical sensor. Reprinted with permission from [91]. 5-HT, 5-hydroxytryptamine; Ag, silver; AgCl silver chloride; Au, gold; DA, dopamine; ECL, electrochemical luminescence; RSD, relative standard deviation.

In another study, an aptamer-based electrochemical sensor was developed to evaluate the neuronal function at the single cell level [91]. This sensor comprised micro-wells, DA aptamers, and co-reactant-embedded polymer dots (Pdots). The sensor’s embedded Pdots provided electrochemical luminescence signals, which served to visualise the electrochemical DA signal (Figure 3a,b). The hybrid structure of this sensor allowed it to capture a single or a small number of differentiated cells inside a micro-well, which then selectively detected the DA released from the captured neurons using the DA aptamer. This sensor exhibited stable cell viability as a cell cultivation and differentiation platform with low cell toxicity. In addition, this sensor demonstrated a low LOD of 53 pM DA (Figure 3c,d) and was capable of evaluating the amount of DA exocytosis.

Nakatsuka et al. reported a DNA aptamer-based nanopipette capable of monitoring the differentiation process of iPSCs into serotonin neurons [92]. This sensor was used to electrochemically detect 5-hydroxytryptamine (5-HT) release from serotonin neurons as differentiated cells. In addition, the nanopipette form of the sensor allowed size exclusion of non-specific proteins in complex culture medium environments, further enhancing the sensor’s 5-HT selectivity. Specifically, this sensor was able to detect 5-HT of less than 3 nM using fast scan CV, which is an excellent sensing capability for DA, similar to the sensing capability of enzyme-linked immunosorbent assay (ELISA). Above all, this sensor was able to detect 5-HT released at the cellular level through the introduction of an aptamer capable of binding specifically to 5-HT.

These studies support that nucleic acid materials can greatly improve electrochemical sensing ability, especially target selectivity, through their specific binding sites. In addition, nucleic acid materials can be combined with other types of biomolecules or sensing probes through chemical conjugation to provide various 3D ligands, which can improve electrochemical sensors’ capabilities.

### 2.3. Carbon Nanomaterial-Based Electrochemical Sensors

Carbon nanomaterials (CNPs), such as graphene and its derivatives, fullerene and carbon nanotubes (CNTs), and nanofibres are composed of chemical structures in which carbon atoms are combined through sp^2^ hybridisation, resulting in the delocalisation of electrons [93]. The intrinsic properties of CNPs depend on their configuration and include good thermal stability, mechanical properties and chemical resistance. Furthermore, due to their structural characteristics, they have good electrical properties, including electron mobility and electrical conductivity. Moreover, CNPs can be synthesised into specific 3D structures, such as CNTs, and sheet forms, such as graphene. In addition, CNPs are biocompatible and can improve cellular functions, for example, cell adhesion and proliferation, making them suitable for stem cell cultivation platforms [94,95,96,97,98,99,100,101,102,103].

In a 2021 article, Castagnola et al. describe a graphene-based electrochemical nanosensor to evaluate neuronal function [104]. This sensor comprised graphene flakes with a 3D arrangement via photolithographic processes. This sensor showed high electrochemically active area enhancement. Specifically, the electrochemically active area of the sensor was about 88 times higher than that of conventional carbon fibre electrodes. The sensor sensitively and selectively detected DA as a target analyte using fast scan CV to evaluate neuronal function; the LOD for DA was calculated to be approximately 364.44 nM. Furthermore, the sensor was demonstrated to discriminate between serotonin and DA. Overall, this sensor’s enhanced electrochemical properties for sensitive and selective sensing of DA suggested that neurogenesis could be monitored in real-time by sensing DA released from neurons in vitro.

**Figure 4:**
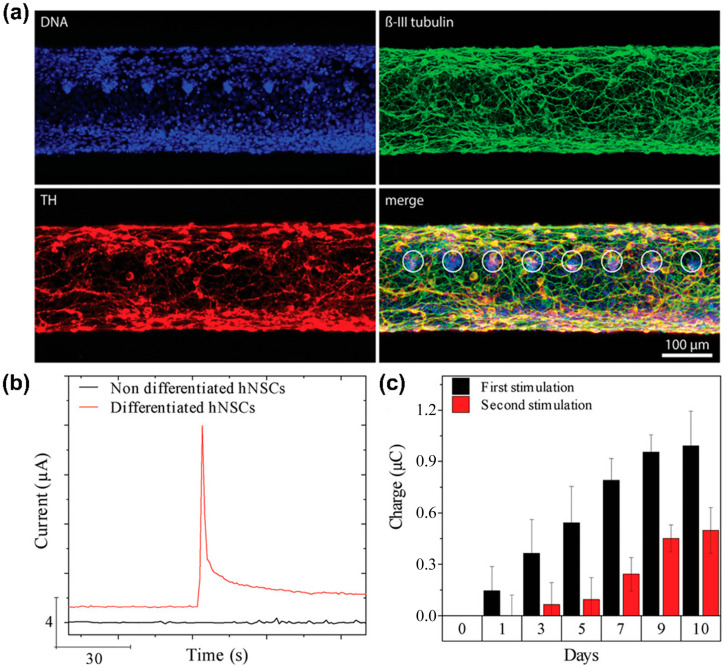
A carbon fibre-based electrochemical sensor for monitoring in vitro neurogenesis. (**a**) Immunocytochemistry images of hNSCs-derived dopaminergic neurons cultured on the sensor. (**b**) Amperometry graph of non-differentiated and differentiated cells after stimulating DA exocytosis. (**c**) Electrochemical current peaks toward DA for time-dependent monitoring of the dopaminergic differentiation on the sensor. Reprinted with permission from [105]. Copyright 2019, Wiley Online Library. DA, dopamine; DNA, deoxyribonucleic acid; hNSCs, human neural stem cells; TH, tyrosine hydroxylase.

Vasudevan et al. [105] developed a nanosensor based on CNPs (Figure 4a). Specifically, this sensor was composed of carbon fibre, which detected DA released from neural stem cell-derived dopaminergic neurons and promoted neurogenesis in vivo based on optogenetics as an optical fibre. To improve the electrochemically active surface area of the sensor, a 15 μm thick polyimide buffer layer was coated onto the surface of a silica-based optical fibre; then, this layer was processed to form of 8 μm thick pyrolytic carbon fibre surrounded by cladding. Subsequently, human neural stem cells (hNSCs) were cultured on the sensor surface and differentiated into dopaminergic neurons to detect DA exocytosis electrochemically using an amperometric method. According to the amperometric results, the electrochemical signal towards DA was not confirmed from undifferentiated cells on the sensor surface. However, a clear electrochemical current peak towards DA was confirmed from the differentiated cells (Figure 4b,c). Moreover, as a result of monitoring the DA signals on the sensor surface during the 10-day differentiation period, it was observed that the DA signal gradually improved according to dopaminergic differentiation. These results suggested that the sensor could monitor dopaminergic differentiation non-invasively.

Similarly, Pham Ba et al. constructed a CNT-based nanosensor to monitor neuronal differentiation [106]. This sensor’s Nafion®-radical layer was composed of CNT transistors and was demonstrated to selectively detect DA in the presence of the interfering molecules acetylcholine and glutamine. In addition, it was confirmed that neuronal cells could be normally attached and cultured on the sensor surface. Moreover, an amperometric response to DA was observed immediately after potassium chloride (KCl) stimulation from neurons cultured on the sensor surface. Furthermore, the sensor obtained different amperometric responses to DA by adding different concentrations of KCl, suggesting that the sensor could discriminate between different degrees of DA exocytosis.

The previously mentioned studies support that carbon nanomaterials, including graphene, carbon fibre, and CNT, can be actively utilised to construct excellent sensing platforms with high electrical properties. In particular, nanosensors based on the graphene family have been demonstrated to monitor neuronal differentiation by sensitively and selectively sensing DA through π-π stacking. Moreover, CNPs’ biocompatibility and high sensing capability make them suitable for stem cell cultivation platforms and non-invasive monitoring of various types of stem cells, such as ESCs and MSCs.

### 2.4. Microfluidic System-Based Electrochemical Sensors

Microfluidic systems refer to systems in which fluid can flow through micro-scale channels fabricated on a substrate [107]. The microfluidic system has the advantage that it can be performed on a single chip under various conditions in a short time using only a small volume of reagents. Due to these structural features, microfluidic systems have been actively applied as biosensors [108,109,110]. Furthermore, given that the microchannel in microfluidic systems enables electrochemical species in the analytes to be confined near the electrochemical electrodes, they are advantageous to electrochemical sensing with high performance [111]. Above all, microfluidic systems can mimic the cellular microenvironment, which can be applied as a stem cell cultivation platform [112].

An example of an electrochemical sensor based on a droplet microfluidic system capable of sensing osteogenic differentiation was described by Fan et al. in 2019 [113]. This sensor could detect the impedance of a single cell; therefore, it was possible to analyse the differentiation of stem cells non-invasively without a label. The sensor detected a difference between the impedance of undifferentiated and differentiated cells. As osteogenic differentiation progressed, the variation in cell impedance decreased. In addition, the average impedance decreased as the differentiation progressed. Conversely, the capacity of the cells analysed on the sensor gradually increased with differentiation. These results were consistent with the fact that calcium ion channels were gradually formed on the cell membrane following osteogenic differentiation.

In 2020, a brain-on-a-chip device based on a microfluidic system capable of analysing neural differentiation was developed [114]. This platform’s design structure included three isolated compartments, suggesting that this structure was suitable for pharmacological manipulations, and a plastic lid and a specific gas supply chamber to build a gas supply system. It was validated that neurons could be cultured and maintained for up to 98 days on the platform. In addition, it was confirmed that neurons were differentiated normally on the device with positive expression of axonal and dendritic markers and that their neuronal networks were formed normally. Furthermore, the spike train tiling coefficient was successfully measured from the neuronal network of neurons differentiated on the platform.

**Figure 5:**
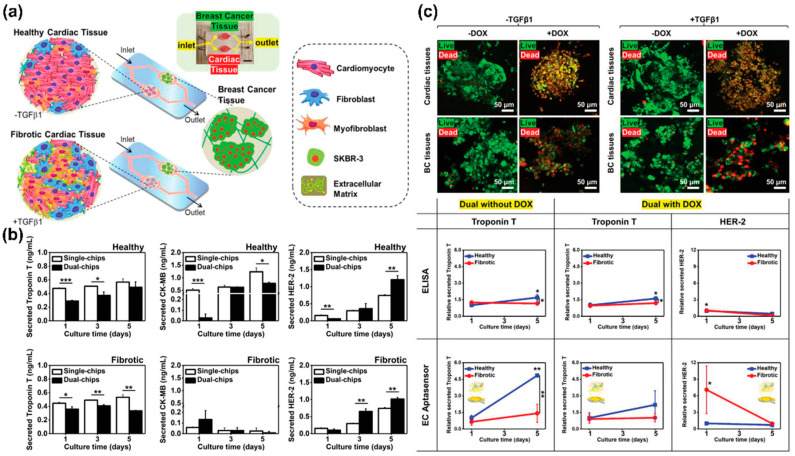
A microfluidic system-based electrochemical sensor for monitoring cardiomyocyte differentiation. (**a**) A schematic illustration showing an aptamer and microfluidic system-based electrochemical sensor. (**b**) Analysis of the important role of interaction between cardiac and heart cancer tissues through biomarkers sensing. (**c**) Monitoring of cardiotoxicity-associated biomarkers using the aptamer and microfluidic system-based electrochemical sensor. Reprinted with permission from [115]. Copyright 2020, Wiley Online Library. DOX, doxorubicin; HER2, human epidermal growth factor receptor 2; TGFβ1, transforming growth factor beta 1.* *p* < 0.05, ** *p* < 0.01, and *** *p* < 0.001.

Another study by Lee et al. described a microfluidic system-based sensing and cultivation platform capable of electrochemically analysing the cellular function of cardiomyocytes differentiated from iPSCs [115] (Figure 5a). Interestingly, the platform developed contained an aptamer and a gold-based microfluidic system. The functionality of cardiomyocytes could be electrochemically monitored by selectively sensing markers, such as troponin T, creatine kinase, and human epidermal growth factor receptor 2; these markers are related to the functionality of cardiomyocytes (Figure 5b). In addition, to investigate the interaction between cardiac and heart cancer tissues cultured on the platform, troponin secreted from each cell was detected after single or dual interaction with the platform on which each tissue was cultured (Figure 5c). Results showed that troponin released from healthy cardiac tissues increased in the single and dual platforms. In contrast, troponin released from healthy tissues on the dual platform was lower than from cells that did not interact with heart cancer tissues. In addition, cell functionality was evaluated electrochemically by measuring biomarkers released from healthy cardiomyocytes and heart cancer cells on the aptamer and microfluidic system-based platforms. The results obtained on the platform were consistent with ELISA results.

Therefore, it can be concluded that microfluidic systems are effective micromaterials for constructing sensing platforms for the non-invasive and label-free monitoring of stem cell differentiation. In particular, microfluidic systems are suitable for application as a stem cell culture platform and can considerably improve the performance of existing electrochemical sensors.

### 2.5. Microelectrode Array-Based Electrochemical Sensors

Microelectrode arrays refer to micromaterials in which multiple micro-scale electrodes are arranged on a single substrate [116]. Microelectrode arrays are based on a 3D structure that improves and increases the electrochemically active area in which analytes participate in the redox reaction, providing a high sensing performance [117]. In addition, considering that a plurality of electrodes is spatially arranged in a microelectrode array, it is possible to analyse signals of cells by position on a single substrate and to analyse single cells and spheroids electrochemically [118].

The literature reports that microelectrode array-based electrochemical sensors that can non-invasively monitor stem cell differentiation have been developed. For example, a microelectrode array that electrochemically monitors cardiomyocyte differentiation has been described [119]. This platform was designed to culture iPSCs for an extended period and to perform qualitative and quantitative analyses of the differentiated cells’ maturity for efficient cardiomyocyte differentiation. The platform was used to monitor the cardiomyocyte differentiation of iPSCs for 119 days using electrochemical impedance spectroscopy. In addition, the effect of 2D and 3D culture environments on cardiomyocyte differentiation was evaluated non-invasively by analysing the differentiated cells’ impedance. The results confirmed that more mature cardiomyocytes were produced in a 3D environment.

Gao et al. [120] decribed a microelectrode array-based sensing platform that monitors neuronal differentiation by electrochemically detecting neurotransmitters released from olfactory bulb neurons. The olfactory bulb neurons were cultured in a microelectrode array located on the corresponding platform; cells were stimulated using glutamate and gamma-aminobutyric acid (GABA) to detect electrochemical signals from the cells. As a result of detecting cells at various concentrations of glutamate and GABA, it was confirmed that cell signals depended on the concentration of the stimulant and that neurotoxicity occurred at high concentrations. The sensing ability of the platform to detect neurotransmitters through GABA stimulation can be used to detect neural differentiation of stem cells non-invasively and label-free.

**Figure 6:**
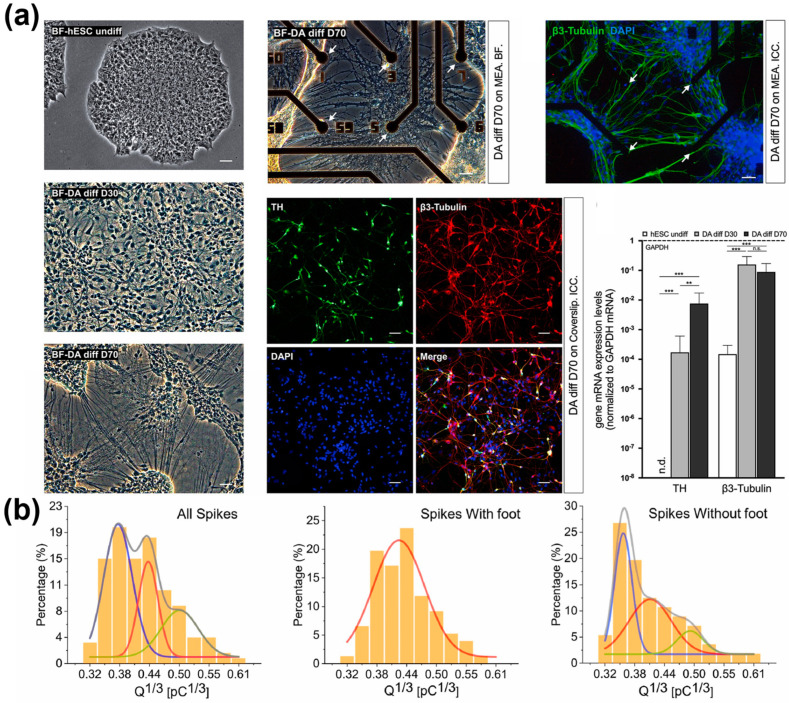
A microelectrode array-based electrochemical sensor. (**a**) Analysis of dopaminergic differentiation of hESCs cultured on the platform. (**b**) Detection of DA exocytosis on the platform. Reprinted with permission from [121]. Copyright 2022, Elsevier. DA, dopamine; DAPI, 4′,6-diamidino-2-phenylindole; GAPDH, glyceraldehyde 3-phosphate dehydrogenase; hESCs, human embryonic stem cells; mRNA, messenger ribonucleic acid; TH, tyrosine hydroxylase. “n.d.”, indicates non-detected; “n.s.”, indicates non-significant; ** *p* < 0.01, *** *p* < 0.001.

Similarly, a sensing platform capable of electrochemically analysing DA exocytosis in dopaminergic neurons differentiated from human ESCs was developed in 2020 [121]. The microelectrode array in the platform was composed of reduced GO and poly(3,4-ethylene dioxythiophene):polystyrene sulfonate (PEDOT:PSS) nanocomposites. Due to the nanocomposites’ excellent electrochemical properties, the device’s LOD for DA was calculated to be 2 nM. Moreover, it could detect DA released from neurons in a location-by-amperometric manner thanks to the intrinsic advantage of the microelectrode array (Figure 6). The platform’s superior dopaminergic sensing ability allowed dopaminergic differentiation to be detected in real-time.

A review of the current literature supports that microelectrode arrays have several advantages as electrochemical sensors. First, the 3D regularly arranged electrodes could considerably improve existing electrodes’ electrochemical sensing ability by increasing the electrochemically active area. Second, the microelectrode array can be used as a stem cell cultivation platform to analyse cells on a single substrate by location. In addition, single cells and spheroids can be effectively detected.

## 3. Optical Sensors

### 3.1. Gold and Silver Nanoparticle-Based Optical Sensors

Nanomaterials, such as AuNPs and AgNPs, have excellent optical properties [122], which are unique depending on their particle size [123,124]. In addition, AuNPs can be modified through chemical conjugation with various other nanomaterials and probes to form new types of hybrid nanomaterials; this allows sensors with higher selectivity and sensitivity to target analytes to be developed [125,126]. In particular, AuNPs can greatly enhance the surface plasmon effect because they are mainly applied to optical sensing Raman spectroscopy methods [127].

Various AuNP-based optical sensors capable of monitoring stem cell differentiation non-invasively and in real-time have been reported. For example, Cao et al. report a gold-based surface-enhanced Raman spectroscopy (SERS) sensor for monitoring osteogenic differentiation [128]. This sensor had a hybrid structure based on AuNPs and nucleic acids and sensitively detectable micro-RNAs (miRs), such as miR-144-3p, associated with osteogenesis. The DNA nucleic acid binds site-specific targets via its complementary interaction. Therefore, hybrid nanostructures in which a specific DNA strand is conjugated on the surface of AuNPs are highly selective. Furthermore, the gold-based nanostructures selectively detected the Raman signals from the target miR. In addition, the probe showed high optical properties and enhanced Raman signals. These advantageous features allowed the sensor to be used in stem cell cultivation and long-term monitoring of their osteogenic differentiation.

In another study, Sun et al. developed a smart gold nanoprobe for detecting alkaline phosphatase (ALP) activity during bone marrow MSCs’ osteogenic differentiation [129]. The smart nanoprobe was designed by decorating the surface of AuNPs with 5-bromo-4-chloro-3-indolyl phosphate (Au@BCIP), which is suitable for use as a SERS nanoprobe. This probe allowed non-invasive and living-cell permeable monitoring of ALP activity with high sensitivity and selectivity. Moreover, this probe could detect a single cell without cell deformation, and the preparation process was simple, so time and effort could be saved compared to conventional methods. Therefore, the non-invasive detection of ALP activity associated with bone disease in vivo models and osteogenic differentiation of bone marrow MSCs can be more fully understood from the perspective of ALP activity.

**Figure 7:**
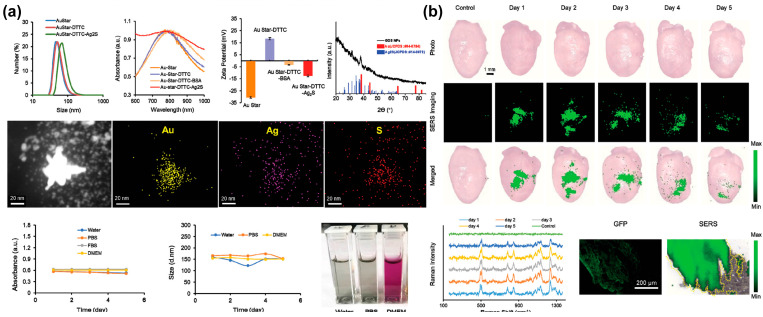
A gold-based NIR sensor for monitoring of chondrogenic differentiation. (**a**) Characterisation of the AuStar-DTTC-Ag_2_S (GDS) nanoparticles. (**b**) Ex vivo Raman imaging of GDS-MSCs in a myocardial infarction model. Reprinted with permission from [130]. Copyright 2020, Wiley Online Library. Ag, silver; Ag_2_S, silver sulphide; Au, gold; AuStar, gold nanostar; BSA, bovine serum albumen; DMEM, Dulbecco’s modified eagle medium; DTTC, 3.3′-diethylthiatricarbocyanine iodide; GFP, green fluorescent protein; MSCs, mesenchymal stem cells; NIR, near-infrared; PBS, phosphate-buffered saline; S, sulphur; SERS, surface-enhanced Raman spectroscopy.

A 2021 article by Hua et al. describes the development of an imaging probe consisting of gold nanostars (AuStar) and silver sulphide quantum dots for labelling and accurately tracking MSCs in a hypodermic and myocardial infarction model with deep tissue penetration [130] (Figure 7). The probe’s AuStar-disseminated tumour cell cluster section enabled high-resolution Raman imaging, effectively delineating stem cells in surrounding normal tissues at a single-cell resolution scale. In addition, the labelling agents were biocompatible and did not alter the MSCs’ biological properties, compensating for existing invasive monitoring methods used for tracking stem cells’ shortcomings.

Lee et al. [131] fabricated magneto-plasmonic nanorods that detected the expression level of miRNA-124 and characterised the neurogenesis of human-iPSC-derived hNSCs in a non-destructive and efficient way. The plasmonic (gold) parts of the nanorod selectively and sensitively recognised target exosomal miRNAs using a molecular beacon (MB), which was on the gold component. The MB and miRNA hybridised to form an MB–miRNA complex with an increased fluorescence signal, increasing the signal-to-noise ratio through the metal-enhanced fluorescence effect. This system was non-destructive and could possibly advance the transplantation of differentiated stem cells.

An ultrasensitive nanosensor consisting of a Au-coated nanopore thin film was reported by Yang et al. in 2021 [132]. This nanosensor was developed for the detection of N27 cells’ Glial cell-derived neurotrophic factor (GDNF) secretion [132]. The GDNF is a small protein that strongly promotes the survival of dopaminergic and motor neurons, and the evaluation of its magnetic stimulation is valuable. Due to the characteristics of gold, the nanosensor’s conversion signal appeared as an optical signal (optical interference fringes) reflected from the nanopore thin film. The optical signal shift occurred because of changes in the effective optical thickness when the GDNF bound to its antibody. The nanostructure helped coat more gold, greatly increasing the sensor’s sensitivity; this was demonstrated by a significantly improved GDNF LOD (2 pg/mL) compared with the rat ELISA kit assay (32 pg/mL). In addition, it was inexpensive, easy to use, and suitable for measuring GDNF secretion with ultra-high sensitivity. Furthermore, it can potentially be used for other highly secreted substances.

In a study on AgNP-based optical sensors, Koh et al. [133] developed a 3D cell culture scaffold and SERS-based biosensor to detect multiple differentiated markers from adipose-derived MSCs. Scaffolds were composed of electrospun nanofibres with hydrogel patterns. Moreover, the sensing scaffolds were coated with AgNPs, which can be conjugated with specific antibodies for SERS analysis. This type of scaffold culture platform successfully supported adipose-derived MSCs’ proliferation and differentiation in osteogenic differentiation media. In addition, the SERS-capture substrate detected various differentiation markers with SERS tags made of Au-Ag alloy nanoboxes. The time-dependent release of three different osteogenic differentiation markers (ALP, osteocalcin, and fibronectin) were detected up to the pg/mL levels without interference or crosstalk for three weeks. Therefore, the platform was sufficiently sensitive to monitor markers during osteogenic differentiation. This platform was suggested to overcome the limitations of existing stem cell differentiation monitoring methods, as it did not require cell pre-processing, enabled continuous analysis with a single platform, and the multi-sensing scaffold could detect various biomarkers.

Similarly, Li et al. [134] developed a SERS sensor for accurate and quantitative detection of DA in blood. The sensor consisted of zipper-like ortho-nanodimers and AgNPs with a uniform 1 nm gap. The AgNPs were electrostatically self-assembled onto a glass slide; then, the complementary DNA of the DA aptamer was bound to the surface of the AgNPs. The SERS probe was synthesised by decorating AgNPs with DA aptamers and the Raman reporter 5,5’-dithiobis-(2-nitrobenzoic acid). When these SERS probes were added to a substrate, they combined with the complementary DNA forming zipper-like ortho nanodimers with a 1 nm gap between the probe and AgNPs on the substrate in a state of equilibrium between electrostatic repulsive force and hybridisation contractility. The uniform gap allowed the SERS sensors to detect DA with ultra-high sensitivity (LOD = 10 aM) while maintaining signal uniformity (relative standard deviation < 5%). Even in a complex serum environment, the sensor maintained excellent validity and stability from 1 pM to 10 nM, which was about two times lower than conventional methods. In addition, monitoring the DA of cells released from living neurons was performed for the first time. This was achieved by introducing a single microfluidic chip containing a 3D cell culture device. The DA quantification in human blood samples showed recoveries ranging from 87.5% to 123.7%. Given the difficulty of DA quantification in complex physiological samples, this SERS sensor may provide a powerful tool for the in vitro investigation of neurological processes and clinical examination of dopaminergic disorders.

Based on the literature reviewed, gold- and silver nanomaterials have excellent optical properties and the advantage of being easily transformable into various 3D nanostructures. In addition, AuNPs can be widely applied to optical sensing methods because of their easy surface modification with other nanomaterials.

### 3.2. Upconversion Nanoparticle-Based Optical Sensors

Upconversion refers to a phenomenon in which external energy is changed into higher energy through a phosphor [135]. Considering that UCNPs show unique optical properties different from conventional phosphors, they have been applied to bioimaging and the optical applications of conventional phosphors. Specifically, UCNPs do not quench and are chemically stable. Furthermore, unlike conventional quantum dots, the maximum emission wavelength does not depend on particle size. In addition, UCNPs can easily emit multi-colour emissions by changing doping materials. In particular, UCNPs doped with lanthanum elements are excited by long wavelengths and have very low cytotoxicity; therefore, they are very useful for use in cell-based sensors [136]. To date, various UCNP-based sensors capable of monitoring stem cell differentiation have been reported in the literature.

For example, Wang et al. [137] developed a multifunctional UCNP capable of real-time detection and control of osteogenic differentiation in MSCs using NIR. The researchers synthesised thulium/erbium-doped core-shell UCNPs coated with mesoporous silica for drug loading and to install photomechanical azobenzene that acted as an agitator. Then, the RGD peptide and matrix metalloproteinase 13 (MMP13) sensitive peptide-black hole quencher-3 group were conjugated to the UCNP surface responsible for cell targeting and detecting differentiation. Finally, icariin, a drug that can induce MSCs’ osteogenic differentiation, was loaded onto the UCNPs to form a nanocomplex. The drug was released from the fabricated UCNP nanocomplexes using NIR light in a controlled way, which was based on trans-azobenzene being converted to a cis isomer under UV and visible light. According to the results of reverse transcription (RT)-PCR, WB, and autonomously replicating sequence (ARS), the UCNP nanocomplex efficiently induced osteogenic differentiation of MSCs under NIR light at a wavelength of 980 nm and successfully detected MMP13 produced by osteogenesis. Therefore, this developed multifunctional UCNP could control the osteogenesis of MSCs and detect cell differentiation in real time, making it a potential tool for progressing regenerative medicine.

Similarly, Yan et al. [138] reported on the controlled osteogenic differentiation of MSCs with a light-responsive nanoplatform to treat osteoporosis (OP). The nanoplatform was a modification of that of Wang et al. described previously. Like Wang et al.’s method, the UCNPs were first doped with thulium/erbium and coated with mesoporous silica. Then photocaged linker 4-(hydroxymethyl)-3-nitrobenzoic acid and polyethylene glycol linker were linked to the surface to conjugate to the cap β-cyclodextrin and the RGD-targeted peptide/MMP13-sensitive peptide-black hole quencher, creating a drug loading nanoplatform. According to the RT-PCR, WB, ALP/ARS/immunofluorescence staining, and ALP activity results, the release of icariin by NIR light at 980 nm induced controlled osteogenic differentiation of MSCs for OP treatment. In addition, MMP13 produced by the MSCs’ osteogenic differentiation cleaved the MMP13-sensitive peptide, removing the peptide-black hole quencher and allowing the UCNPs to fluoresce; this allowed real-time detection of osteogenic differentiation. The results of haematoxylin and eosin, Masson’s trichrome, immunohistochemical, tartrate-resistant acid phosphatase, and toluidine blue staining of a femoral terminal section showed that significant bone remodelling had occurred in the OP rat model. This study’s results suggested that the synthesised UCNP nanoplatform enabled remote control and real-time detection of osteogenic differentiation for OP treatment by NIR and could be a potential alternative to current OP treatment.

**Figure 8:**
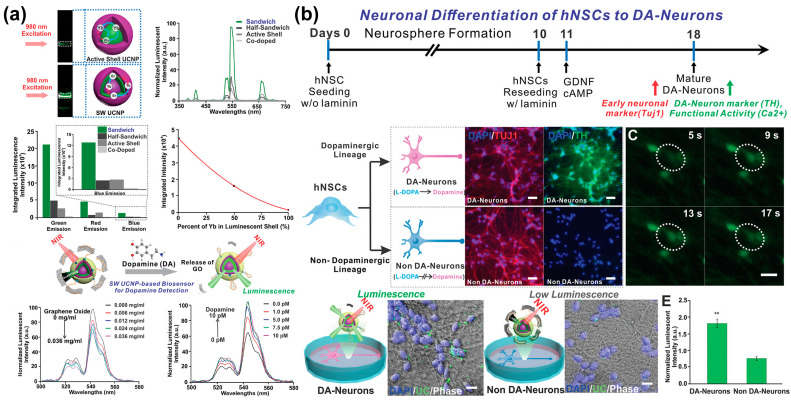
Upconversion nanoparticle-based optical sensor capable of monitoring neurogenesis. (**a**) Upconversion luminescence profiles and analysis of the operating mechanism and characteristics of UCNPs for each condition. (**b**) Monitoring of neuronal differentiation of hNSCs using the UCNPs-based optical sensor. Reprinted with permission from [139]. Copyright 2019, Wiley Online Library. cAMP, cyclic adenosine monophosphate; DA, dopamine; GDNF, neurotrophic factor; GO, graphene oxide; hNSCs, human neural stem cells; NIR, near-infrared; UCNPs, upconversion nanoparticles. ** *p* < 0.01.

Non-destructive stem cell differentiation control and monitoring using UCNPs has also been studied for neural differentiation from MSCs. However, conventional UCNPs have shortcomings, such as low emission intensity due to undesirable energy transfer paths. Low power density excitations can minimise detrimental energy reverse transitions and produce bright visible emissions. Therefore, Rabie et al. [139] developed a core–shell–shell sandwich-structured UCNP with enhanced luminescent output relative to conventional UCNPs. This core–shell–shell UCNP was then used to construct a biosensor to detect DA released from stell cell-derived dopaminergic neurons (Figure 8). This UCNP detected DA released in vivo during the differentiation of stem cells into specific neurons at the single cell level in a highly selective, real-time, and non-invasive manner, with a sensitivity of at least three times higher than similarly designed systems. This sensor was demonstrated to detect DA at low concentrations. The developed NIR-based neurotransmitter detection method has significant potential for the diagnosis of diseases related to neurodegenerative diseases and stem cell treatment strategies.

The literature identified several UCNPs with high optical properties and low cytotoxicity. Moreover, UCNP-based sensors can monitor stem cell differentiation in real-time, non-invasively, without a label. In addition to cell imaging, UCNPs are potential drug delivery systems to control stem cell differentiation. 

### 3.3. Autofluorescence-Based Optical Sensors

Various biomaterials derived from cellular organisms have autofluorescence properties, which allows them the potential to be used in biosensors for invasive and label-free sensing to obtain information about cells and tissues [140]. Furthermore, autofluorescence techniques do not require treatment or fixing of specimens and can be performed in real-time. In addition, autofluorescence-based sensors can be applied to monitor stem cell differentiation with invasive optical sensing methods, such as Raman spectroscopy and NIR, because autofluorescence can indicate a specific cellular component [141,142]. Raman-based sensing applied with autofluorescence is especially advantageous to analyse information about intracellular dynamics, which can be utilised to investigate the intracellular changes during stem cell differentiation [103].

**Figure 9:**
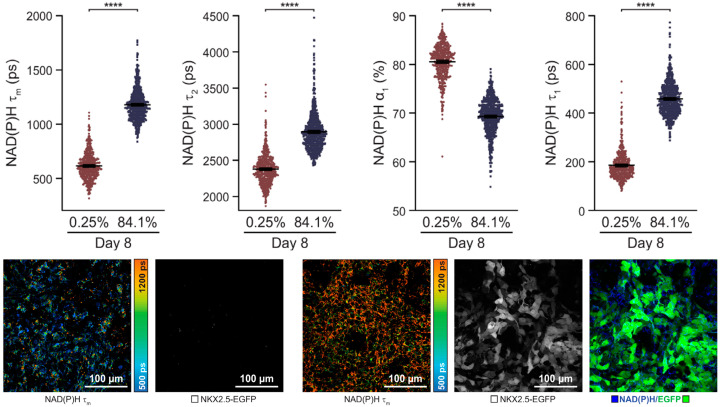
An autofluorescence imaging for monitoring of cardiomyocyte differentiation. Single-cell quantitative analysis of NAD(P)H during 8 days of the differentiation period. Reprinted with permission from [143]. Copyright 2019, Wiley Online Library. EGFP, enhanced green fluorescent protein; NAD(P)H, reduced nicotinamide adenine dinucleotide (phosphate); NKX2.5-EGFP, homeobox protein NKX2.5-EGFP.**** *p* < 0.0001.

A label-free autofluorescence-based imaging system combining optical metabolic modelling with quantitative image analysis was developed by Qian et al. [143] for monitoring human PSC (hPSC) differentiation into cardiomyocytes (Figure 9). This study was based on the fact that hPSC-derived cardiomyocytes undergo significant metabolic changes during differentiation. Specifically, the amount or ratio of oxidised flavin adenine dinucleotide (FAD) and reduced nicotinamide adenine dinucleotide (phosphate) (NAD(P)H), both autofluorescence metabolic materials, is influenced by the cellular conditions and differentiation and can be imaged to collect metabolic information at the single cell level. Furthermore, the ratio of NAD(P)H to FAD provides information about the relative oxidative state of the cells. Therefore, cardiomyocytes differentiated from different hPSC lines were visualised with NAD(P)H and FAD autofluorescence probes. According to the autofluorescence imaging on the eighth day, the intensity of NAD(P)H autofluorescence differed depending on the differentiation efficiencies. In addition, the cardiomyocytes and non-cardiomyocytes showed different autofluorescence intensities. For instance, NAD(P)H and FAD fluorescence after 8 days was exhibited in 84.1% of the differentiated cells, compared with 0.3% in undifferentiated cells.

In another study, Suhito et al. [144] reported on an autofluorescence-integrated Raman mapping analysis for label-free monitoring of adipogenic differentiation. Raman mapping analysis has the critical issue of long detection time, which results in cell apoptosis. To address this issue, these researchers developed a novel optical sensing method that enabled the rapid and non-destructive analysis of adipogenesis. The authors confirmed that the lipid droplets present in adipocytes were identified with the developed autofluorescence-integrated Raman sensing method; the Raman scattering of lipid droplets was aroused at 2850–2855 cm^−1^. In addition, this method was utilised in the large-scale sensing analysis of multiple cells in culture plates by obtaining Raman mapping images at low magnification. Moreover, the analysis required a very short time (<20 min) and could scan 440 × 330 μm area per mapping image. Furthermore, the authors analysed in-batch and batch-to-batch variations of adipogenic differentiation throughout the autofluorescence-Raman imaging.

Similarly, Li et al. showed a label-free autofluorescence sensing system capable of monitoring of neurogenesis [145]. The optical sensor was based on tetrapod-shaped ZnO (t-ZnO) microparticles capable of label-free monitoring of neuronal differentiation. Specifically, this sensor formed 3D scaffolds that analysed DA released from neurons embedded on the surface using autofluorescence imaging. Interestingly, t-ZnO nanoparticles with four hexagonal arms were biocompatible and autofluorescence materials that fluoresced under UV light because they contained anion vacancies. The nanoparticles’ autofluorescence was demonstrated to be very sensitive to hole scavengers, which was used for quantitative DA analysis. Furthermore, nanoparticles’ autofluorescence acted as a quencher for the autofluorescence of the t-ZnO nanoparticles. Due to its 3D structures with high surface area and autofluorescence, the t-ZnO nanoparticles-based sensor showed a high sensing performance toward DA (LOD: 0.137 μM). Furthermore, DA was selectively datable in the presence of interfering molecules, including citric acid, glutamine, ascorbic acid, glucose, KCl and calcium chloride.

Autofluorescence-based optical sensing techniques were reported as suitable for non-invasive, non-destructive, and label-free monitoring of stem cell differentiation. Chronic lymphocytic leukaemia-derived autofluorescence could be utilised to analyse the biological changes in stem cell differentiation. Furthermore, most autofluorescence is biocompatible, which allows stem cells to be stably cultured and differentiated on the sensor for long time periods.

## 4. Conclusions and Future Perspectives

This review summarised recent progress in nano-biosensors for non-invasively monitoring of stem cell differentiation. Various nano- and micromaterials, such as AuNPs, AgNPs, UCNPs, autofluorescence probes, nucleic acids, microfluidic systems and microelectrode arrays, were reviewed and compared. Furthermore, their advantageous use to improve the biosensors’ performance, including sensitivity and selectivity, for monitoring stem cell differentiation was appraised. In the case of electrochemical sensors, the electrochemically active surface area was found to be a crucial parameter; this is because the redox reaction of target analytes occurs on the electrode surface via electron transfer. Moreover, various nanomaterials were identified that could be applied to improve the electrochemically active surface. Furthermore, specific micro-scaled systems were identified that could be utilised to enhance the advantage of existing nanomaterials, providing more sensitive and selective sensing capabilities toward target molecules. The literature scrutinised supported that many nanomaterials have been applied in optical sensing to enhance the optical signal intensity to target analytes specifically or to make the analysis process simpler and faster. In addition to excellent sensing capabilities, various nano-biosensors have functioned as stem cell cultivation platforms by providing cell-friendly surfaces. In conclusion, electrochemical or optical nano-biosensors capable of monitoring stem cell differentiation in a non-invasive, non-destructive, and label-free sensing system could be used to control stem cell differentiation and develop practical and efficient stem cell therapies.

**Table 1:** Nano-Biosensors for Non-Invasive Monitoring of Stem Cell Differentiation.

Method	Material and Technique	Advantages	Limitations	Target/Sensitivity	Differentiation	Ref.
Electrochemical sensing	AuNPs	▪ High biocompatibility towards hESC growth and adhesion ▪ Label-free identification of differentiated cells	▪ Insufficient sensitivity	Cells/12,500 cells	Epithelial differentiation	[59]
	AuNPs-nanoarray	▪ Non-destructive and real-time sensing system for monitoring of osteogenic differentiation ▪ Providing topographical cues favourable for stem cell differentiation	▪ Low sensing performance at early differentiation stages	ALP/0.03 unit/mL	Osteogenic differentiation	[73]
	AuNPs-RGD-Fe_3_O_4_	▪ Capable of regulation of mechanotransduction-mediated stem cell differentiation ▪ In-situ time-resolved monitoring of osteogenic differentiation	▪ Difficult analysis of early differentiation	ALP/-	Osteogenic differentiation	[74]
	AuNPs	▪ Simultaneous electrochemical monitoring and kidney organoid generation on-a-chip ▪ Electrical signal interpretation in multiple detection of kidney ▪ organoid on-a-chip	▪ Difficult to identify detailed cell subtypes	Cells/21,363 cells	Kidney organoids	[75]
	Aptamer-CPNTs	▪ Highly sensitive and selective detection of DA ▪ Non-destructive monitoring of exocytotic DA release from living neurons	▪ Potential colouration effect and its poor stability	DA/100 pM	Neuronal differentiation	[89]
	Peptide-Oligonucleotide	▪ Non-destructive detection of cardiomyogenic differentiation ▪ Applicable to label-free drug screening	▪ Insufficient selectivity for applications in cell culture environments	cTnI/0.42 pg/mL	Cardiomyocyte differentiation	[90]
	Aptamer-Pdots-microarray	▪ High throughput DA sensing at the single cell level ▪ Imaging of exocytotic DA release from living neurons	▪ High variations in DA signals	DA/53 pM	Neuronal differentiation	[91]
	Aptamer	▪ Based on a nanostructure suitable for excluding non-specific proteins as interfering molecules ▪ Suitable to track in situ neurochemical flux in the complex cell media	▪ Difficult sensor operation	5-HT/< 1 nM	Neuronal differentiation	[92]
	Graphene-	▪ Simultaneously detection of DA and serotonin ▪ Potential for multi-site neurotransmitter mapping with high spatial and temporal resolution	▪ Platform not proven applicable to cell culture	DA/61.67 nM	Neuronal differentiation	[104]
	Pyrolytic carbon fibre	▪ Real-time monitoring of exocytotic DA release ▪ Multifunctional platform applicable to stem cell-based sensing and therapeutics	▪ Insufficiently proven sensor selectivity	DA/-	Neuronal differentiation	[105]
	Carbon nanotube	▪ Label-free and non-invasive evaluation of antipsychotic drugs on DA release from neuronal cells	▪ Insufficient selectivity toward DA	DA/<10 nM	Neuronal differentiation	[106]
	Microfluidics	▪ Label-free and non-invasive impedance measurements of single cells	▪ Insufficient for practical applications in terms of throughput	-	Osteogenic differentiation	[113]
		▪ Compartmentalised the neuronal culture into three distinct but axonally connected networks ▪ Mimicking local and circuitry functionality of brain tissue	▪ Includes complicated devices and procedures	-	Neuronal differentiation	[114]
		▪ Non-invasive monitoring of cell-secreted multiple biomarkers in response to chemotherapeutic drugs ▪ Contains 3D cardiac and BC spheroid-based microtissues with mechanical properties mimicking individual native tissues	▪ Short-term (<6 days) analysis of differentiation	Troponin T and HER-2/<0.1 pg/mL	Cardiomyocyte differentiation	[115]
	Microelectrode array	▪ Long-term (>100 days) monitoring of cardiomyocyte differentiation with 3D cultivation system	▪ Signal variations during monitoring differentiation	E4031/<1 nM	Cardiomyocyte differentiation	[119]
		▪ In vitro multi-site and long-term monitoring of bioelectrical activity changes in the neuronal network ▪ Cross-correlation analysis between channels capable of evaluating the connection status in the neuronal networks	▪ Insufficient biological validation	Glutamate and GABA/100 nM and 50 nM, respectively	Neuronal differentiation	[120]
	Microelectrode array-rGO	▪ Stable monitoring of neuronal differentiation from long-term cultured neurons ▪ Quantitative analysis of different types of DA vesicular exocytosis	▪ Insufficient selectivity toward DA	DA/2 nM	Neuronal differentiation	[121]
Optical sensing	AuNPs-DNA	▪ Long-term (>21 d) sensing of biomarkers involved in osteogenic differentiation ▪ Label-free monitoring of osteogenic differentiation	▪ Potential side effects of cellular uptake of AuNPs	miR-144-3p/13.6 aM	Osteogenic differentiation	[128]
	AuNPs-BCIP	▪ Label-free monitoring of osteogenic differentiation	▪ Potential side effects of cellular uptake of AuNPs	ALP/1.0 U/L	Osteogenic differentiation	[129]
	AuStar-DTCC-Ag_2_S	▪ Depth-independent and high-resolution stem cell-tracking strategy ▪ Real-time sensing of in vivo functionality of stem cells	▪ Potential side effects of cellular uptake of AuNPs	Cells/<100 cells	Adipogenic differentiation Osteogenic differentiation Chondrogenic differentiation	[130]
	Au-Ni nanorods	▪ Highly selective detection of stem cell differentiation ▪ Applicable to in vitro and in vivo stem cell differentiation modulation	▪ Potentially undesired effects of magnetic fields on cell behaviour	miR-124/<1 pM	Neuronal differentiation	[131]
	AuNPs-nanopore	▪ Cost-effective and sensitive sensing method for detection of neuronal differentiation	▪ Signal variations during monitoring differentiation	GDNF/<2 pg/mL	Neuronal differentiation	[132]
	AgNPs	▪ Label-free sensing system for sensing multiple osteogenic differentiation-involved markers. ▪ 3D culture system integrated-sensing system	▪ Signal variations during monitoring differentiation	ALP, OC, and FN/-	Osteogenic differentiation	[133]
	AgNPs-Aptamer	▪ Ultrasensitive and reliable detection of exocytotic DA release from living neurons ▪ 3D culture system integrated-sensing system	▪ Insufficient biological validation	DA/10 aM in PBS and 10 fM in serum	Neuronal differentiation	[135]
	UCNP	▪ Simultaneous differentiation control and sensing of osteogenic differentiation ▪ Label-free detection of osteogenic differentiation	▪ Potential cytotoxicity by photothermal effect	MMP13/-	Osteogenic differentiation	[137]
		▪ Simultaneous differentiation control and sensing of osteogenic differentiation ▪ Applicable to in vitro and in vivo stem cell differentiation modulation	▪ Potential cytotoxicity by photothermal effect	MMP13/-	Osteogenic differentiation	[138]
		▪ Monitoring of neuronal differentiation at single-cell level ▪ Real-time analysis of the function of DAnergic neurons	▪ Potential cytotoxicity by photothermal effect	DA/<1 pM	Neuronal differentiation	[140]
	Autofluorescence	▪ Label-free monitoring of cardiomyocyte differentiation ▪ Can predict the cardiomyocyte differentiation efficiency early in the differentiation process	▪ Potential phototoxicity during imaging procedures	FAD and NAD(P)H/-	Cardiomyocyte differentiation	[143]
		▪ Non-destructive and large-scale analysis (440 × 330 μm area) of adipogenic differentiation ▪ Rapid visualisation (<20 min) of the cell morphology and cytosolic microstructure	▪ Difficult to apply to multiple-cell analysis	Lipid droplets/-	Adipogenic differentiation	[144]
	t-ZnO	▪ Utilisable directly without complicated probe immobilisation or modification ▪ High resolution detection of DA release from neural tissue	▪ Insufficient lifetime for living tissue cultivation	DA/0.137 μM	Neuronal differentiation	[145]

Note: 3D, three-dimensional; 5-HT, 5-hydroxytryptamine; AgNPs silver nanoparticles; Ag_2_S, silver sulphide; ALP, alkaline phosphatase; AuNPs, gold nanoparticles; Au-Ni, gold-nickel; AuStar, gold nanostar; BC, breast cancer; BCIP, 5-bromo-4-chloro-3-indolyl phosphate; CPNTs, carboxylated polypyrrole nanotubes; cTnI, cardiac troponin I; DA, dopamine; DAnergic neurons, dopaminergic neurons; DNA, deoxyribonucleic acid; DTCC, disseminated tumour cell clusters; E4031, N-[4-[1-[2-(6-methylpyridin-2-yl)ethyl]piperidine-4-carbonyl]phenyl]; FAD, flavin ade-nine dinucleotide; Fe_3_O_4_, iron (II, III) oxide; FN, fibronectin; GABA, γ-aminobutyric acid; GDNF, glial cell-derived neurotrophic factor; HER-2, human epidermal growth factor receptor-2; miR, microRNA; MMP13, metalloproteinase 13; NAD(P)H, nicotinamide adenine dinucleotide (phosphate); OC, osteocalcin; Pdots, polymer dots, RGD, Arg-Gly-Asp ligand; rGO, reduced graphene oxide; t-ZnO, tetrapod zinc oxide; UCNPs, upconversion nanoparticles.

## Data Availability

Not applicable.
